# Development of nucleic acid aptamer-based lateral flow assays: A robust platform for cost-effective point-of-care diagnosis

**DOI:** 10.7150/thno.56471

**Published:** 2021-03-05

**Authors:** Tao Wang, Lanmei Chen, Arpitha Chikkanna, Suxiang Chen, Isabell Brusius, Nabayet Sbuh, Rakesh N. Veedu

**Affiliations:** 1 Centre for Molecular Medicine and Innovative Therapeutics, Murdoch University, Perth 6150, Australia.; 2 Perron Institute for Neurological and Translational Science, Perth 6009, Australia.; 3 Guangdong Key Laboratory for Research and Development of Nature Drugs, School of Pharmacy, Guangdong Medical University, Zhanjiang 524023, China.

**Keywords:** Aptamer, lateral flow assay, point-of-care, biosensor, sandwich assay, competitive assay.

## Abstract

Lateral flow assay (LFA) has made a paradigm shift in the *in vitro* diagnosis field due to its rapid turnaround time, ease of operation and exceptional affordability. Currently used LFAs predominantly use antibodies. However, the high inter-batch variations, error margin and storage requirements of the conventional antibody-based LFAs significantly impede its applications. The recent progress in aptamer technology provides an opportunity to combine the potential of aptamer and LFA towards building a promising platform for highly efficient point-of-care device development. Over the past decades, different forms of aptamer-based LFAs have been introduced for broad applications ranging from disease diagnosis, agricultural industry to environmental sciences, especially for the detection of antibody-inaccessible small molecules such as toxins and heavy metals. But commercial aptamer-based LFAs are still not used widely compared with antibodies. In this work, by analysing the key issues of aptamer-based LFA design, including immobilization strategies, signalling methods, and target capturing approaches, we provide a comprehensive overview about aptamer-based LFA design strategies to facilitate researchers to develop optimised aptamer-based LFAs.

## 1. Introduction

The effective detection of hormones, proteins and microorganisms in clinical samples, or contaminants in food or environment is crucial for disease control and public health management. Although various currently used analytical techniques including high-performance liquid chromatography (HPLC), mass spectrometry, ELISA assays allow highly sensitive and specific detection, their application depends heavily on trained personnel and sophisticated instrumental facilities 1. Consequently, efforts have been made to develop simplified point-of-care test (POCT) devices featured by ease of use, rapid response, high affordability, and low analyte volume requirement 2. Lateral flow assay (LFA), also known as immunochromatographic assay or strip test, is one of the most investigated POCT technologies 3, 4.

**Figure 1:**
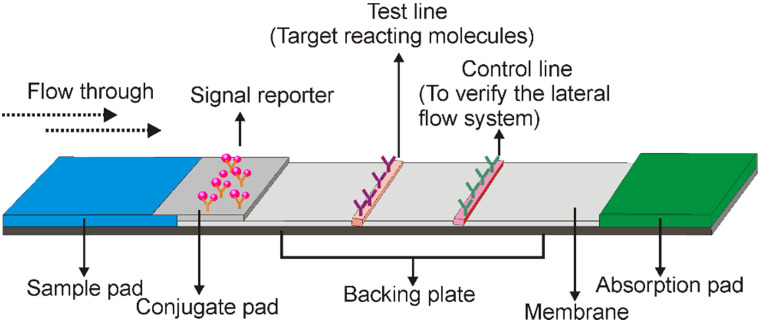
** Schematic illustration of a typical lateral flow assay strip.** It includes five components that are assembled on a paper strip. After adding liquid samples to the sample pad (blue), it migrates from the conjugate pad, test line, and control line to the absorption pad. Target molecules are captured with a colour indicator in the test line, and the lateral flow system is validated by the control line.

A typical LFA consists of three elements, the recognition element, reaction element, and signal transduction element. All of these elements are assembled on a paper strip including five components: the sample pad, conjugate pad, membrane, absorbent pad, and backing plate 5. As shown in Figure 1, while the backing plate provides support for strip assembling, all the other components are overlapping (2-3 mm) with each other sequentially to ensure that the liquid solution migrates through the LFA strip. In general, the sample pad is made of cellulose or glass fibre, to transport the sample under test to the following components of the strip 5. The recognition agent (e.g. antibody)-conjugated reporter molecules (e.g. gold nanoparticles) are loaded on the second conjugate pad, which is commonly made of glass fibre, cellulose, polyesters or other materials being able to rapidly hydrate and release the labelled recognition agent upon meeting liquid samples. When the target analytes in the sample meet the recognition agent/reporter complex, a new complex (target/recognition agent/reporter) is created which then directed over to the test line. The test line is usually lineated on the nitrocellulose membrane and immobilized with target reacting molecules (e.g. aptamer, antibody or complementary oligonucleotides), to react with either the target analyte or the recognition agent and create a signal (the type of signal depends on the nature of the signalling elements) for qualitative or semi-quantitative analysis 3. As the recognition agent/reporter complex is normally present in excess amount on the conjugate pad, the unbound recognition agent/reporter complexes can move further to the control line, which contains a capturing agent to recognize the unbound recognition agent/reporter molecules to validate the performance of the LFA system. The adsorbent pad is located at the end of the strip which serves as a blotting paper to maintain the flow direction of the liquid over the membrane 3.

The prototype design of LFA was first reported in 1956 by Plotz and Singer 6, which later gained explosive attention from the 1980s when the urine-based pregnancy test became commercially available 7. Indeed, as a type of disposable paper assay with a user-friendly format, LFA fulfils the framework of ASSURED (affordable, sensitive, specific, user-friendly, robust, equipment-free and deliverable) for POCT evaluation proposed by the World Health Organization 8. Consequently, the LFA industry rapidly progressed over the past three decades. As of 2018, the LFA industry has reached ~ 6.0 billion US dollars worldwide, and is expected to reach ~8.7 billion USD by 2023, with a compound annual growth rate of 7.7% 9. As a key component, the recognition element of LFA has long relied on antibodies. Nevertheless, the integration of antibodies is hampered by a variety of factors, mostly due to its protein nature, such as irreversible temperature-induced denaturation, prolonged preparation time, difficulty in chemical modification, and importantly, inaccessible for nonimmunogenic and toxic molecules 10. For improved LFA design, alternative recognition agents are of tremendous importance.

The rapid development in aptamer technology provides an opportunity to circumvent the obstacles faced by the antibody-based LFAs. Aptamers, first described in 1990, are short single-stranded DNA or RNA sequences that can bind to targets with high specificity and affinity 11 because of their unique ability to adopt three-dimensional shape in solution. Differing from traditional antibodies, aptamers have some obvious advantages including low batch-to-batch variation, prolonged shelf-life, low/no immunogenicity, and freedom to incorporate chemical modifications for enhanced stability and binding properties 12, 13. Over the past decades, different types of aptamer-based LFAs (Apt-LFAs) have been exploited for target detection in various fields such as disease diagnosis 14, 15, environmental science 16, and food industry 17. However, the practical application of Apt-LFA is still lagging behind that of antibody-based tests, with no Apt-LFA test kits being commercially available 18. Development of highly sensitive and specific Apt-LFA depends on the improvement in some key technique issues, including the method for target and aptamer immobilization, recognition agents, and the signalling system. In this work, firstly, we provide an overview of Apt-LFAs, regarding the design strategies and recent advances. Then, via investigating the existing obstacles, opinions and suggestions for improved Apt-LFA development are provided.

## 2. Application of aptamers in LFA development

### 2.1 Wide applications of Aptamers for target detection

**Figure 2:**
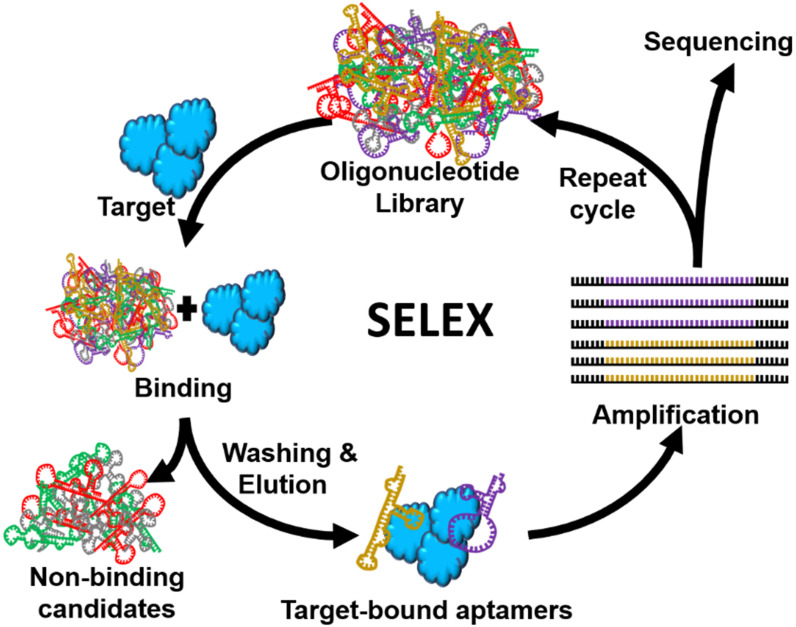
** Schematic illustration of SELEX protocol for aptamer identification.** SELEX consists of a very defined iterative procedure, i.e. library preparation, target and library incubation, bound/unbound sequence separation, elution of target bound sequences, PCR amplification, and single-stranded DNA separation (for DNA aptamer) or transcription (for RNA aptamer). Differing from traditional antibodies, the development of aptamers is performed *in vitro.*

Aptamers, also known as “chemical antibodies”, can be used for nearly all antibody-based applications 19, 20. However, compared with antibodies which are mainly used for recognizing biological materials such as proteins, aptamers show great flexibility to recognize virtually all substances including small molecules (e.g. antibiotics, metal ions), viruses and even whole cells 21, 22. This is because unlike antibodies, the development of aptamers is conducted *in vitro* via a procedure called systematic evolution of ligands by exponential enrichment (SELEX) (Figure 2), to identify DNA or RNA aptamer sequences that are able to recognize the target of interests from a randomised (~ 10^16^ members) nucleic acid library 11. With such a high diversity, aptamers can be developed for any target. Importantly, SELEX procedure can help developing aptamers that bind to low-molecular-weight compounds, which are not readily conducive to antibody generation. Taking fungicide malachite green as an example, because no antibody is available for such small molecular antibiotics, the detection of it has depended on time-consuming and expensive HPLC and liquid chromatography-mass spectrometry (LC-MS) assays 23 until the development of a 38-mer malachite green aptamer in 1999 24, based on which, for the first time, an Apt-LFA for simplified malachite green residue detection became available 25. Similarly, Apt-LFAs for the detection of various non-immunogenic targets such as apple stem-pitting Virus (ASPV) 26 and organophosphorus pesticides 27 have also been established.

### 2.2 Flexible LFA design

Aptamers composed of natural DNA or RNA nucleotide sequences are vulnerable to the nucleases, particularly RNA aptamers which can be degraded in serum in seconds 19. On the bright side, aptamers are accessible to incorporate a large panel of chemical modifications including 2'-O-methyl (2'-OMe), 2'-fluoro (2'-F), 2'-amino (2'-NH2), and locked nucleic acid (LNA) 28, for enhanced stability and binding affinity. In addition, by sequentially linking two different DNA aptamers (with the same target), a tandem aptamer structure with improved binding capacity can be obtained as reported for VEGF protein targeting aptamer 29. Likewise, the unique polymeric nucleic acid structural features of aptamers make it possible for developing novel LFA platforms such as split aptamer-based LFA and target-induced dissociation (TID) mediated LFA (Section 3). Furthermore, the nucleic acid nature of aptamers also enables to develop simplified LFA design via adding a short nucleic acid sequence region (e.g. 5'-AAAAAA-3'; complementary capture sequence of an additional sequence, e.g. 5'-TTTTTT-3') at the end of the detection aptamer to serve as a universal capturing agent for the control line design 30.

## 3. Existing Apt-LFA formats

The antibody-based LFA was introduced in clinical practice long before the invention of aptamer technique. Given the similarities between aptamer and antibody in terms of the tertiary structure-based target recognition, knowledge acquired from antibody-based biosensor design could be useful for Apt-LFA development. Over the past decades, various Apt-LFA approaches including sandwich formats, competitive formats, and other novel methods based on the structural and functional features of aptamers have been introduced, as described below.

### 3.1 Sandwich Apt-LFA

The sandwich assay method is the most used Apt-LFA, especially for the detection of large molecular weight analytes like proteins 30. In a typical sandwich Apt-LFA, after loading samples to the sample pad, the target molecules are firstly captured by a detection aptamer (often conjugated with a reporter molecule) resulting in the formation of a reporter-conjugated aptamer/target complex on the conjugate pad. Upon migration to the test line, the target molecule in the complex can be recognized by a secondary affinity agent and forms a sandwich structure with the target molecules in the middle (between the reporter-conjugated aptamer and the secondary affinity agent). By far, three different types of sandwich Apt-LFAs have been proposed.

#### 3.1.1 Sandwich Apt-LFA using dual aptamers

**Figure 3:**
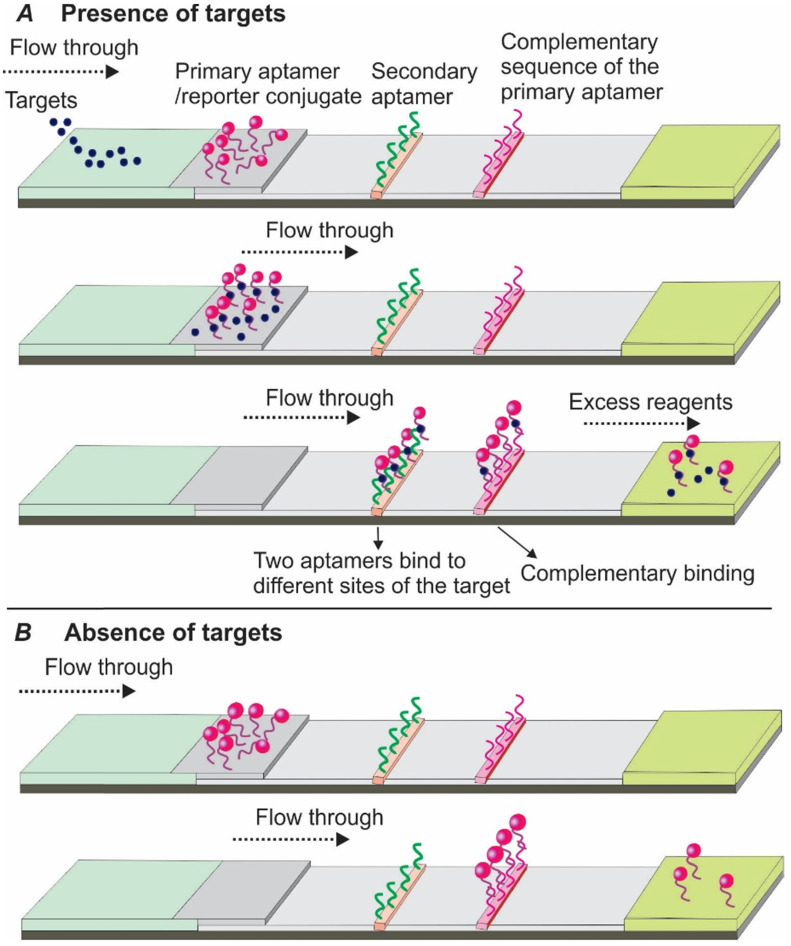
** Schematic illustration of the dual aptamer-based sandwich Apt-LFA.** (A) In the presence of target molecules, the targets firstly bind to the detection aptamer and create a target/aptamer/reporter complex. When passing through the test line, the target/aptamer/reporter complex is then captured by a capture aptamer immobilized on the test line and results in the aggregation of the signal reporter, while the unreacted complex and the free aptamer/reporter are captured by a complementary oligonucleotide sequence immobilized on the control line to validate the LFA system; (B) In the absence of target molecules, the aptamer/reporter conjugate passes beyond the test line and is captured by specific complementary oligonucleotide probe on the control line. In both cases, the excessive reagents are absorbed by the absorption pad.

In 2009, Xu and colleagues introduced a sandwich Apt-LFA for thrombin detection 30, with a format identical to the conventional antibody-based LFAs. As shown in Figure 3, a pair of aptamers targeting different sites of the thrombin molecule were employed. Firstly, one of the aptamers, namely the detection aptamer, was conjugated to the gold nanoparticles (AuNPs) via thiolation and loaded onto the conjugate pad, serving as the recognition element. The second aptamer was biotinylated and immobilized onto the test line via streptavidin-biotin binding (streptavidin was pre-coated onto nitrocellulose membrane), serving as a capturing aptamer. After thrombin containing samples are loaded and migrated to the conjugate pad by capillary action, a thrombin/AuNP-conjugated detection aptamer complex was formed. The complex then continued to migrate along the strip to the test zone, where the complex was captured by a capture aptamer, and resulted in the aggregation of AuNPs (show a characteristic red colour, Figure 3A). The excess complexes then passed beyond the test line and then captured by fixed oligonucleotide sequences complementary to a specific region of the detecting aptamer on the control line resulting in another red band. In the absence of thrombin, a distinct red band was shown only on the control line (Figure 3B).

Later, following a similar procedure, different sandwich Apt-LFAs were reported, to detect various types of targets ranging from proteins 31, 32, viruses 33 to whole cancer cells 34. Indeed, with two different aptamers immobilized on the conjugate pad and test line respectively to recognize different sites of the target analytes, many of the reported assays demonstrate high specificity and sensitivity in both target spiked buffer and clinical samples (Table 1).

#### 3.1.2 Sandwich Apt-LFA using a combination of aptamer and antibody

One of the major shortcomings of sandwich Apt-LFA is the difficulty in identifying dual aptamers targeting different sites of a target molecule, especially for small molecules with limited binding domains for aptamer recognition. To address this issue, in addition to improving the SELEX procedure for selecting aptamer pairs, the combined use of antibody and aptamer has been exploited. In 2017, this strategy was explored by Minagawa and colleagues for salivary α-amylase (sAA) detection 35. In this work, using a 75-mer DNA aptamer as the detection aptamer (conjugated with AuNP for signal readout), and a commercial anti-sAA antibody loaded on the test zone for sAA/AuNP-conjugated aptamer complex capturing, the authors developed an aptamer/antibody sandwich LFA allowing specific sAA detection in 0.1% (v/v) human saliva. However, the incorporation of the unstable and expensive antibodies contradicts the advantage of the integration of aptamers in LFA. Therefore, the application and further commercialization of such a format may not be feasible.

#### 3.1.3 Sandwich Apt-LFA using split aptamers

**Figure 5:**
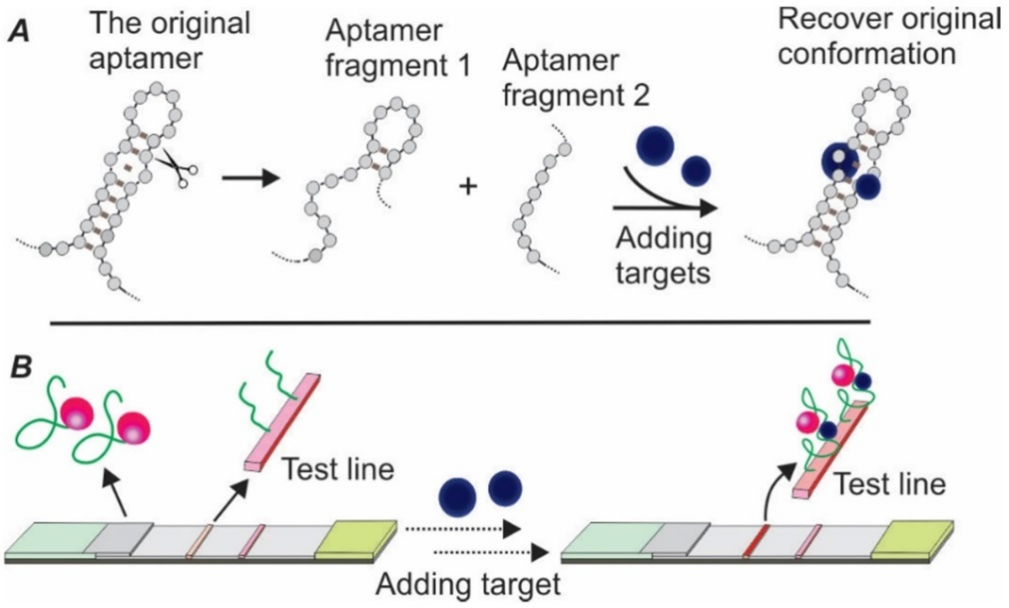
** Schematic illustration of target-induced aptamer reassembling and split aptamer-based LFA.** (A) In the presence of target molecules, two fragments of an aptamer could regain the original structure of the aptamer; (B) Overview of the split aptamer-based LFA.

To address the issue of lacking dual aptamers for LFA development, the concept of split aptamers was introduced by exploiting the structural flexibility of aptamers 36, 37. The mechanism of split aptamer design is based on the target-induced reassembling of aptamer fragments. As shown in Figure 5A, in the presence of target molecules, two separate aptamer fragments could regain the three-dimensional structure and recover the affinity property of the parent aptamer. By conjugating one fragment of the aptamer to a signal reporter (e.g. AuNPs) and immobilizing the other fragment onto the test zone (serving as capturing agent), a sandwich LFA could be created (Figure 5B).

The first split aptamer-based biosensor was developed in 2009. In this work, via designing separate segments of two aptamers, Zuo and colleagues developed an effective electrochemical assay for cocaine and ATP detection 38. The idea of using split aptamers for biosensor development soon became popular, with various types of targets, including thrombin (exciton energy transfer-based fluorescent sensing) 39, 17β-estradiol (absorption-desorption colourimetric detection) 40, and D-vasopressin (electrochemical biosensor) 41. In 2016, a sandwich LFA based on split aptamers was pioneered for ATP detection 42. In this work, the authors designed a pair of split oligonucleotides based on a previously reported ATP aptamer. While the signalling element was produced via labelling one of the aptamer fragments onto AuNPs and loaded onto the conjugate pad, the test zone was prepared by adding the second aptamer split onto the nitrocellulose membrane via streptavidin-biotin interaction. The developed Apt-LFA displayed a linear concentration-signal response within a wide range from 0.5 nM to 5 mM. When tested with solutions spiked with other nucleotides, including UTP, CTP and GTP, non-specific detection was not observed.

Notably, the application of split aptamers has been especially useful in the area of small molecule detection. As previously discussed, many of the small molecules are either not compatible with antibody development or lack a second binding site for dual aptamer identification. Theoretically, a split aptamer pair can be designed by dividing any aptamer into two separate fragments. However, split aptamers generated from different cutting sites of the parental aptamer could display vastly different binding affinities 43. Although investigations have been conducted to elucidate the influence factors for optimised split aptamer design 36, 43, a general principle is still not available. As suggested, to ensure the efficiency of split aptamer-based biosensors, the split sites have to be experimentally tested 43.

In conclusion, although both combined aptamer/antibody and split aptamer strategies have been exploited in recent years, dual aptamer-based sandwich LFA is preferred for highly-sensitive and specific LFA development. Further progress in aptamer identification techniques including the usage of efficient and diverse initial libraries (e.g. G-quadruplex library) 44, next-generation sequencing (NGS) based candidate identification 45, as well as rational counter selection strategies (e.g. using aptamer binding sites inhibitors) 46 would help to facilitate the development of high-quality dual aptamer-based LFA.

### 3.2 Competitive Apt-LFA

Competitive assay (also termed inhibition assay) represents another option when dual aptamers for a target are not available. Two types of competitive assays have been mostly exploited, with immobilizing either target molecules or oligonucleotides partially complementary to the aptamer to the test one to compete for the binding of target molecules to the aptamer. To our knowledge, there is only one exception reported where the aptamer was directly immobilized on the test line 47.

#### 3.2.1 Competition between the target molecule and a complementary sequence for aptamer recognition

**Figure 6:**
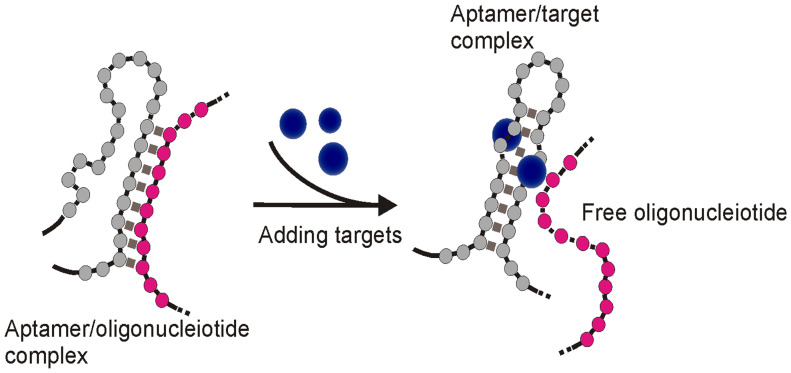
** Schematic of target induced dissociation (TID).** Upon binding to target molecules, aptamer sequences may undergo conformation change to accommodate the target molecule, which results in the dissociation of complementary sequences originally bound to aptamer regions undergoing structural change.

**Figure 7:**
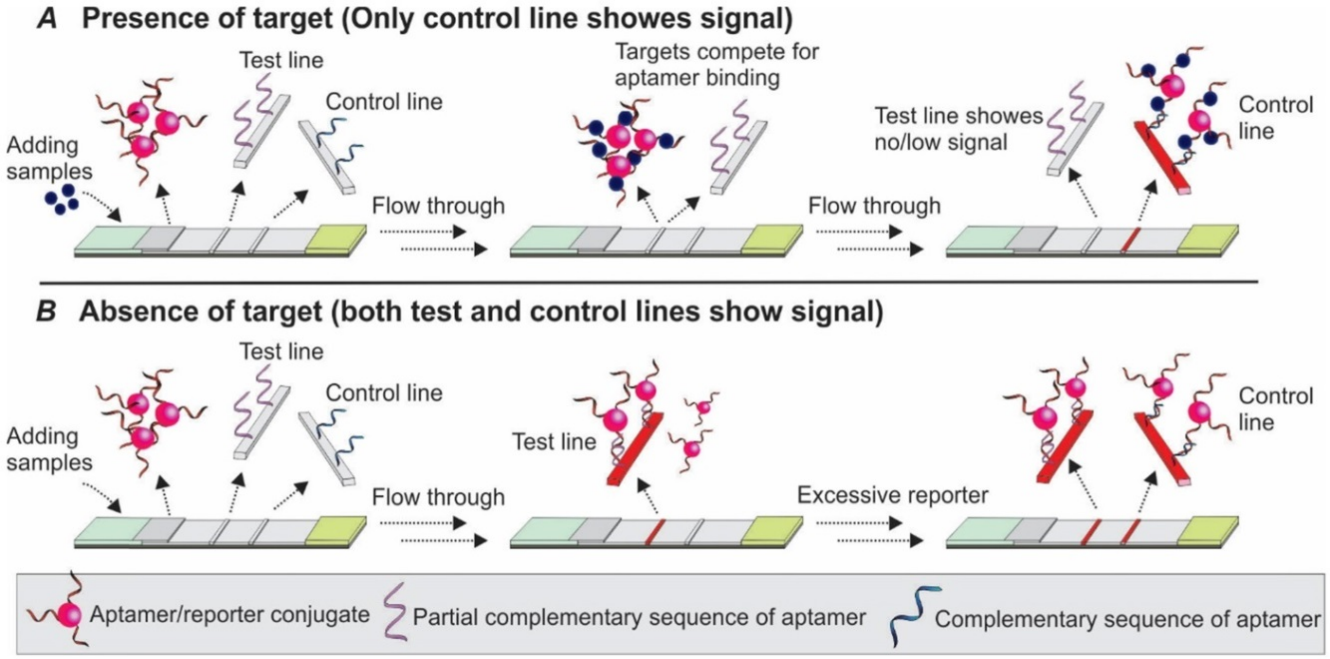
** Competitive Apt-LFA with a partial complementary sequence of the aptamer on the test line.** (A) In the presence of target molecules, the binding of target molecules to the aptamers inhibits the binding of aptamers to their partially complementary sequence and results in no/low signal on the test line; (B) In the absence of target molecules, the aptamer is captured by its partial complementary sequence and shows signals on the test line. In both cases, the control line is labelled with a complementary sequence of the aptamer to validate the flow system.

Upon binding to targets, an aptamer undergoes conformational changes to accommodate the target molecules by forming special structures including hairpin, stem-loop or G-quadruplex 48. When the structural change occurs in certain regions of the aptamer sequence, the Watson-Crick base pairing between the aptamer and its complementary sequence could be interrupted, and result in the target-induced dissociation (TID; Figure 6) 49. Theoretically, by immobilizing an oligonucleotide partially complementary to the aptamer sequence on the test zone, the existence of target molecules in a sample could be detected by monitoring the binding between the immobilized complementary sequence and the aptamer. As shown in Figure 7, in a typical TID-based competitive Apt-LFA, the conjugate pad is labelled with aptamer-conjugated reporters (e.g. AuNPs), which migrate after resuspension towards the test and control lines. In the presence of the target analyte, the target could compete with the complementary oligonucleotide sequence for aptamer recognition on the test line, and display a weaker or no signal (Figure 7A). In contrast, in the absence of the target analytes, the aptamer/reporter complex could be easily captured by the complementary sequence (immobilized on the test line) and display a strong band on the test line (Figure 7B). Notably, differing from the sandwich assays, in a typical competitive assay, an increased intensity of the band at the test line reflects a decreased amount of target molecules in the sample.

In 2011, Wang and colleagues developed a competitive Apt-LFA based on the quantum dot (Qdot, a fluorescent reporter) technique for rapid and highly sensitive detection of ochratoxin A (OTA) 50. The competition strategy was designed by labelling: 1. a poly-A tagged OTA aptamer/Qdot complex to the conjugate pad, and 2. a ssDNA sequence (DNA1) partially complementary to the OTA aptamer to the test zone. After the OTA/aptamer/Qdot complex was formed on the conjugate pad and reached the test zone, OTA in the complex started to compete with the DNA1 immobilized on the test zone for aptamer binding, and demonstrated a signal negatively correlated with the OTA concentration of the sample. As an assay control, a poly-T probe (DNA2) was pre-loaded on the control zone to capture the poly-A tagged detection aptamers existing in either the aptamer/Qdot or OTA/aptamer/Qdot complexes. Featured by a limit of detection (LOD) of 1.9 ng/mL (comparable to ELISA and fluorescence polarization immunoassay), the whole assay could be completed in 10 min. Later, the application of this type of competitive Apt-LFA was extended to the detection of antibody-inaccessible small molecules such as mercury 51 and aflatoxin B1 (AFB1) 52 from river water, wine, herb and corn samples, with the range of LOD from 0.1 ng/mL to 1 ng/mL (Table 1).

Although TID-based competitive Apt-LFA approach demonstrates great potential, it should be noted that it is hard to achieve high sensitivity and specificity. This is mainly because of the difficulty in the complementary oligonucleotide design. As reported, to obtain an ideal complementary sequence, the designed oligonucleotide has to be tested experimentally to ensure the dissociation constant is within a certain range (determined by both the aptamer and the target) 53. For example, a short sequence with a lower degree of complementarity to the detection aptamer may result in a weak hybridization and thereby false-positive signals (weak or no band on the test line in the absence of the target); in contrast, a long oligonucleotide sequence with much stronger binding to the detection aptamer than the target may cause a false-negative signal (strong band on the test line in the presence of the target) 54. Generally, to maintain a proper competition, a complementary sequence with the same or slightly lower affinity to the aptamer than the target is desired 54. Apart from the difficulty in designing complementary sequences, controlling the optimised kinetics of the sequence hybridization and liquid flow represents additional obstacles for TID-based Apt-LFA. As reported, a minimum of 500 seconds is generally required for effective oligonucleotide hybridization 55, while in a typical LFA, the sample solution may pass the immobilized sequences in seconds. To solve these problems, another mode of competitive assay, exploiting the competition between the target molecules in sample solution and the target molecules immobilized on the membrane, has been more commonly exploited.

#### 3.2.2 Competition between the target in sample and target immobilized on the test zone for aptamer recognition

**Figure 8:**
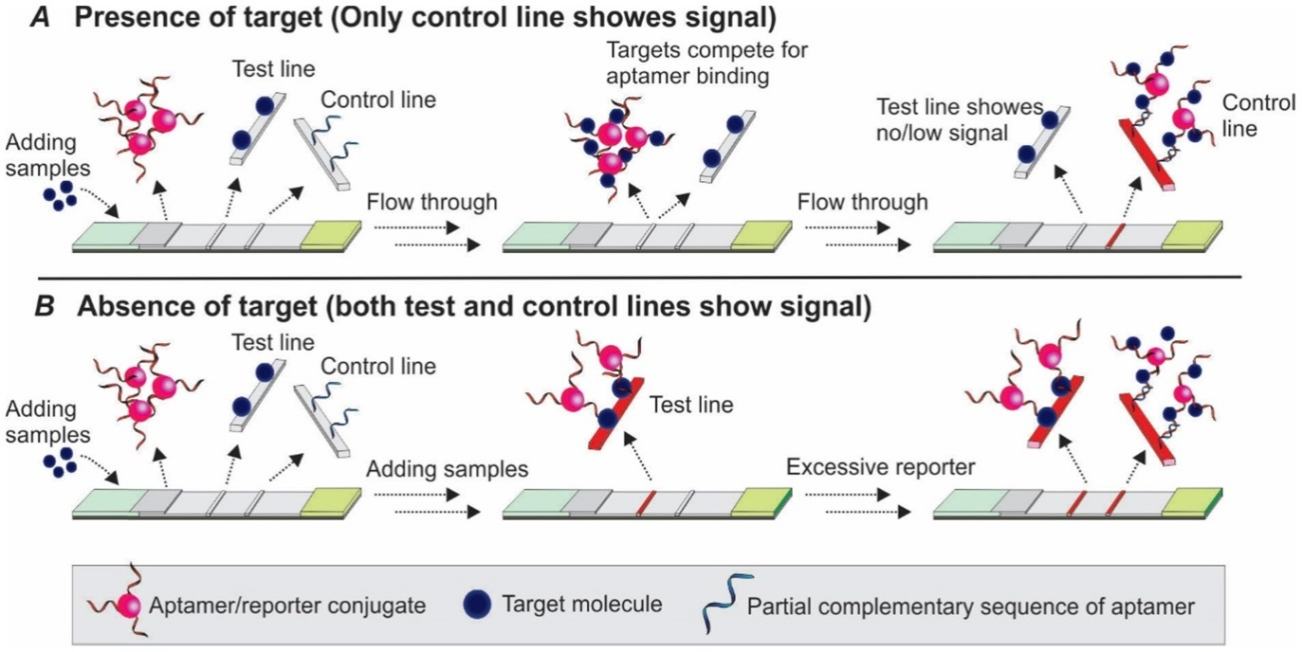
** Competitive Apt-LFA with target molecules on the test line.** (A) In the presence of target molecules, the target molecules in the sample and test line compete to bind the aptamer which results in no/low signal on the test line; (B) In the absence of target molecules, the aptamer is captured by the target molecules on the test line and shows signals. In both cases, the control line is labelled with a complementary sequence of the aptamer to validate the flow system.

As shown in Figure 8A-B, the basic principle of this type of competitive LFA is based on the competition between the target molecules in the sample and target molecules immobilized on the test line surface for aptamer binding. After the target containing samples are loaded to the sample pad and bound to the aptamer/reporter complex on the conjugate pad, the target/aptamer/reporter complex could continue to travel to the test line, where the same target molecules are pre-loaded. As the targets in the samples and targets on the test zone display comparable binding affinity to the detection aptamer, a competition between them takes place, and results in the signal change in accordance with the amount of target molecules in the sample. Similar to the TID-based competitive assay, as the increase in target concentration in the sample results in less free aptamers to bind to the immobilized target molecules on the test zone, a reduced signal will be observed in the test line (Figure 8A). Based on this principle, in 2016, Jauset-Rubio and colleagues developed an Apt-LFA for β-conglutin detection 56. Firstly, thiolated β-conglutin aptamers-conjugated AuNPs were prepared and loaded to the conjugate pad. Then, recombinant β-conglutin and ssDNA sequences fully complementary to the detection aptamer were added onto the test line and control line, respectively. As reported, with an assay time of merely 5 min, the authors achieved a LOD of 55 pM - 10 mM. Furthermore, subsequent modification via recombinase polymerase assisted signal amplification, the LOD could be reduced dramatically to 9 fM, similar to a previously reported sandwich Apt-LFA 17. As for the control line, since the immobilized 94 nt ssDNA sequence was fully complementary to the detection aptamer, it displayed a much higher binding affinity than that of the β-conglutin molecule to the aptamer. Upon passing the control line, both the unbound aptamer/AuNP complex and the aptamer/AuNP/β-conglutin complex could be captured for LFA validation.

It should be noted that the immobilization of expensive and unstable proteins on LFA membrane compromises the merit of Apt-LFA development. Consequently, this method is preferred for detecting low cost and highly stable small molecule compounds. Even so, immobilization of small molecules on membranes may lead to the conformational change of the molecule and results in the failure of aptamer-target recognition 57. As a result, the binding capacity of the detection aptamer to the immobilized target molecules must be experimentally tested 58. To solve this problem, in a pioneer study, Lars Kaiser and colleagues developed a cross-recognition aptamer-based competitive LFA for small molecule - ampicillin - detection 58. Via an *in silico* analysis of the sequence homologies between ampicillin and C-reactive protein aptamers, the authors first identified an aptamer sequence displaying affinity to both ampicillin and C-reactive protein. Then, via immobilizing C-reactive protein onto the test line, and using the cross-recognition AuNP-conjugated aptamer for ampicillin detection, a competitive Apt-LFAs was developed. After incubation with samples and adding the mixture to the sample pad, in cases that no ampicillin was presented, the AuNP conjugated detection aptamer would bind to the immobilized C-reactive protein on the test line and show a clear signal. In contrast, if ampicillin was present in the sample, a competition took place and resulted in a decreased signal on the test zone. Although a relatively low LOD was recorded in this work, it provides an alternative strategy for single aptamer-based small molecule detection. Indeed, via introducing a big molecule (e.g. protein), the problem associated with small-molecule immobilization could be effectively solved. Importantly, although a computer-aided cross-recognition aptamer design was used in this work, theoretically, identification of cross-recognition aptamers simultaneously recognizing two (or more) targets is also achievable via designing proper counter selection during the SELEX procedure (only keeping aptamers that bind to both targets during selection while discarding the sequences that bind to none or only one of the targets). This cross-recognition aptamer-based competitive LFA therefore represents a versatile and transferable general approach for small molecule detection.

#### 3.2.3 Other competitive Apt-LFAs

**Figure 9:**
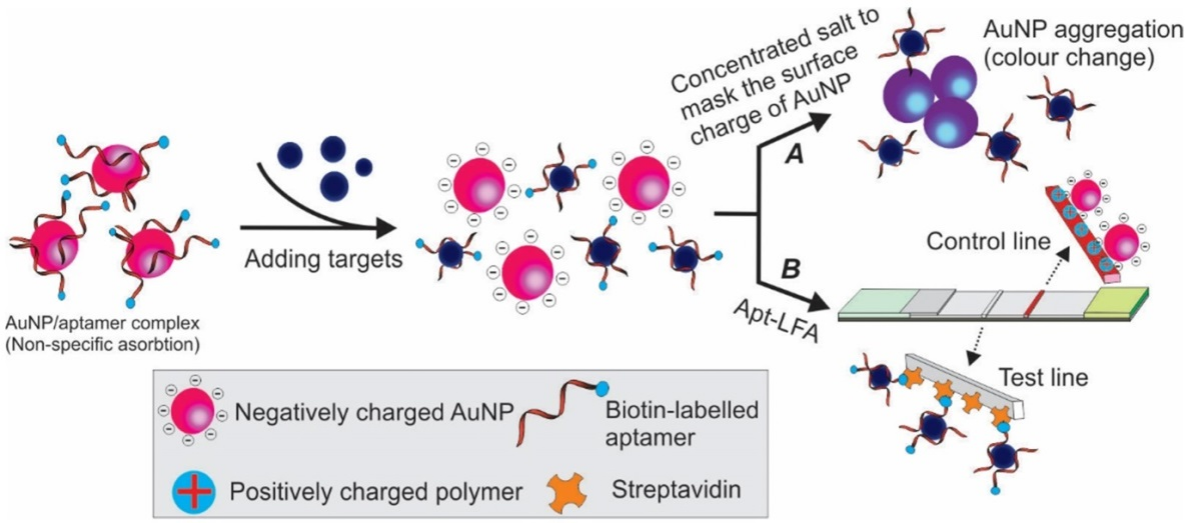
** Schematic illustration of adsorption-desorption mediated Apt-LFA.** After adding target-containing samples, the binding of target/aptamer results in the dissociation of aptamer from the negatively charged AuNP. (A) By adding concentrated salt, the surface charge of AuNP could be masked and result in aggregation and colour change; (B) A lateral flow strip is prepared with streptavidin on the test line and a charged polymer on the control line. In the presence of target molecules, the dissociation of biotin labelled aptamer from the reporter results in no/weak signal on the test line, while the negatively charged free reporter could be captured by positively charged polymer on the control line to validate the assay system.

In addition to the commonly used competitive Apt-LFA as discussed above, in recent years, via exploiting the unique structure of aptamers, other competitive Apt-LFAs have also been practised. The adsorption-desorption colourimetric method previously used for aptamer-based in-solution tests represents a promising strategy 59. As shown in Figure 9A, after binding to target molecules, the weak conjugation between aptamers and AuNPs (via physical adsorption) could be interrupted, resulting in the release of naked AuNPs. Similar to ethanol-based nucleic acid precipitation, in high concentration salt (e.g. NaCl, NaAc) solution, the surface charge and electrostatic repulsion of naked AuNPs could be masked and lead to their aggregation 60. Since the colour of AuNP is strictly decided by its size (and the size of the aggregates), a clear red to grey/blue change could be observed 61. Via this principle, Derosa and colleagues introduced a single aptamer-based LFA for HER2 detection, with streptavidin and cationic charged PDDA polymer on the test line and control line, respectively 62. As demonstrated in Figure 9B, firstly, a weak non-covalent aptamer/AuNP conjugation was created by incubating 5'-biotin-conjugated HER-2 aptamers with citrated AuNPs (citrate was pre-coated to provide a negative charge to AuNP). Then the complex was added to the sample for a short incubation, followed by applying the solution on the sample pad of the LFA. When HER2 protein was present in the sample, the binding between HER2 and aptamers could lead to the release of the aptamers/HER2 complex from AuNPs and result in the release of free AuNPs (due to TID). When the loaded solution migrates to the test line, the biotinylated aptamer/HER2 complex could be captured by the immobilized streptavidin. As no AuNPs were attached to the aptamers, no colour signal could be detected. As the free AuNPs were covered by negatively charged citrate, they could be captured by the cationic charged PDDA polymer on the control line to validate the assay system. In contrast, when HER2 was absent from the sample, the biotin-aptamer/AuNP complex could be easily captured by streptavidin molecules on the test line and display a red signal. Although representing a simple method for single-aptamer-based LFA, the success of this method relies heavily on the quality of the aptamer/AuNP complex. In addition to titrating the optimum density of aptamers to AuNPs, the adsorption force of aptamers to AuNPs needs to be optimized. This is because while a too faint adsorption may result in spontaneous release of aptamers and result in false positive readout, a too strong adsorption could prevent the release of aptamers from AuNP even in the presence of target molecules and result in false-negative results 63.

**Figure 10:**
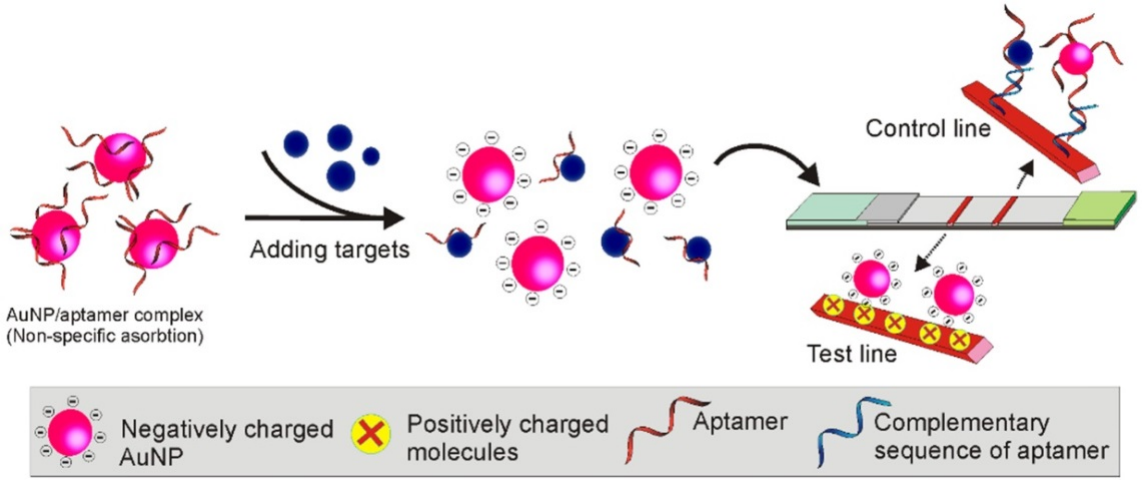
Schematic illustration of adsorption-desorption based Apt-LFA.

Following the same principle, Dalirirad and colleagues developed a modified adsorption-desorption colourimetric LFA for cortisol (a stress biomarker) detection from sweat 64. As shown in Figure 10, similar to that in Derosa's study, the aptamer/AuNPs conjugation (weak non-covalent binding) was prepared by incubating cortisol aptamers and AuNPs at room temperature for 2 hours. However, rather than capturing biotinylated aptamers by streptavidin, the test line was immobilized with cysteamine to capture the citrate stabilized AuNPs. After incubating the aptamer/AuNP complex with the sample, in the presence of cortisol, the conformational change of aptamers, caused by the binding between aptamer and cortisol, could interrupt the weak aptamer/AuNP conjugation and result in the release of the free AuNPs. After loading to the sample pad, followed by passing through the cysteamine (positively charged) immobilized test line, the naked AuNPs (precoated with negatively charged citrate) in the mixture could be captured and display red colour. In contrast, when cortisol was not present in the sample, the aptamer masked AuNP could not be recognized by the cysteamine and no colour could be detected on the test line. Similar to many other Apt-LFAs, the control line was labelled with an oligonucleotide sequence complementary to the aptamer to capture either the free aptamers or AuNP-conjugated aptamers for system control. As known, a significant disadvantage of the conventional competitive Apt-LFA lies in its signalling mode. Obviously, the occurrence of a new signal on the test zone in the presence of the target is preferred for visual detection over the gradually faded signal as observed with conventional competitive LFA. This study provides an example to produce a competitive Apt-LFA with a readout method similar to the sandwich LFA (occurrence of a new signal on the test zone in the presence of the target).

**Figure 11:**
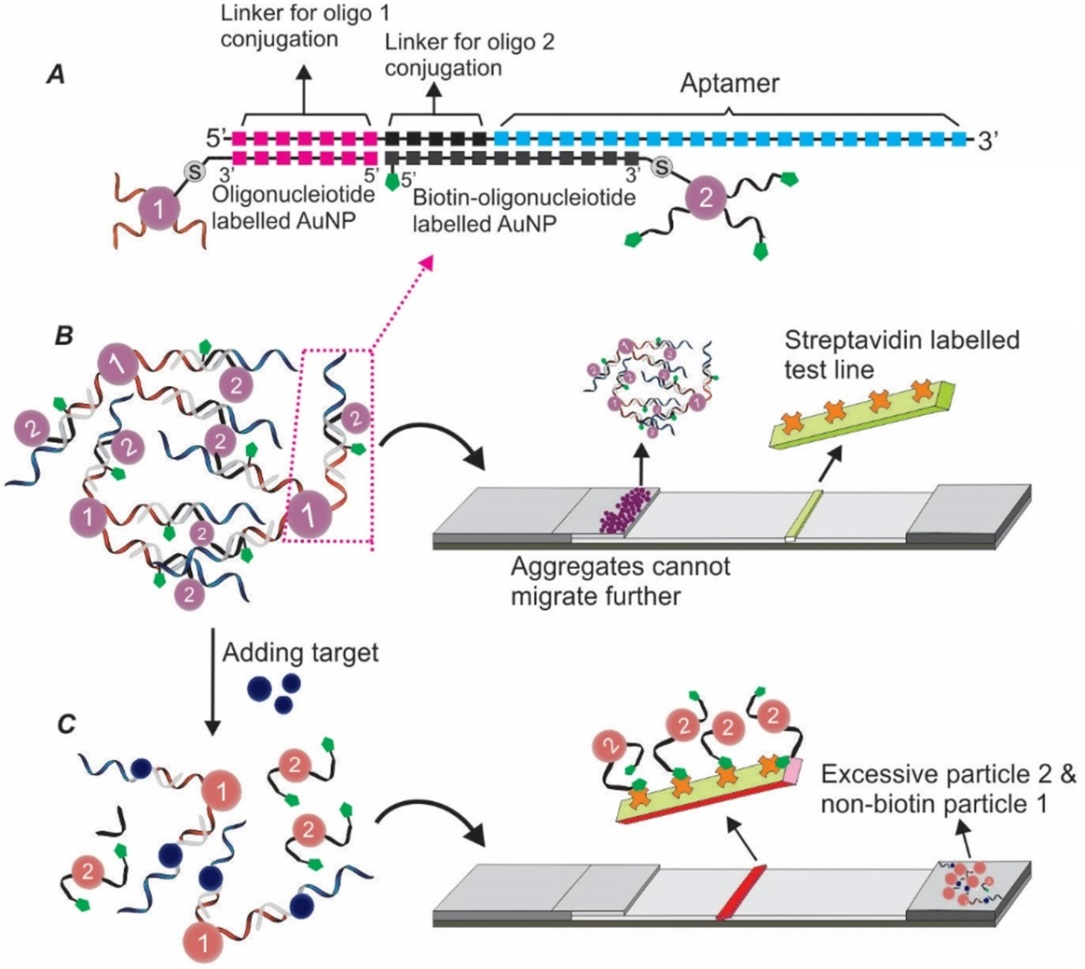
TID based competitive Apt-LFA via exploiting the crosslink mediated reporter aggregation.

Over the past decades, considerable efforts have been made to solve the signalling drawback of conventional competitive LFAs. For example, in 2006, Li's group developed a unique single-aptamer-based LFA for the detection of small molecules adenosine and cocaine 65. As shown in Figure 11, firstly, the authors extended an adenosine aptamer by synthesising additional sequences complementary to two short thiolated ssDNA sequences. Then, through conjugating the two short sequences to AuNP surfaces, AuNP aggregates were prepared via crosslinking the aptamers and these two short sequences. As the addition of the adenosine led to the formation of adenosine/aptamer complex (dis-assembling the AuNP aggregates) and the release of the biotinylated short sequence/AuNP conjugates, a distinct red colour could be detected in the test zone (labelled with streptavidin). As the original aggregates could not migrate along the membrane because of the massive molecular weight, a dark colour could be observed on the boundary of the conjugate pad and the nitrocellulose membrane, serving as a test control.

**Figure 12:**
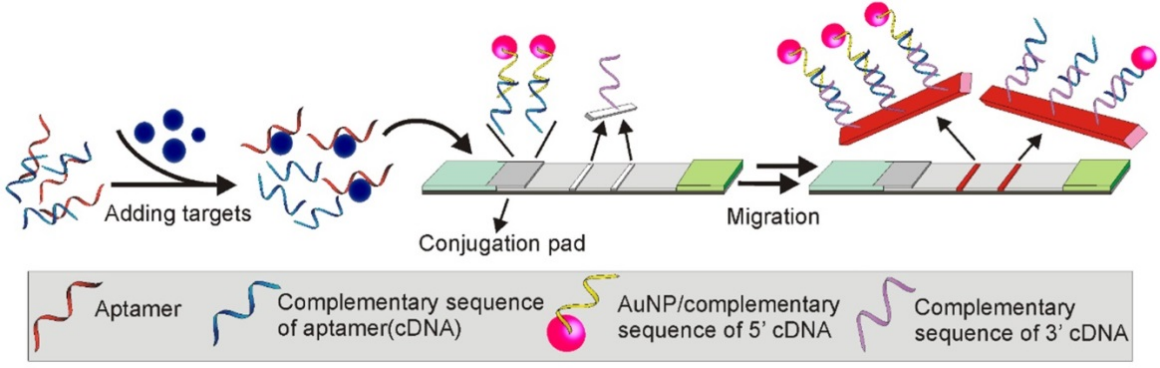
** Competitive Apt-LFA combined with nucleic-acid based sandwich assay.** The aptamer was hybridized with a partially complementary sequence (cDNA). In the presence of targets, the structural change of the aptamer induced by target molecules results in the release of the complementary sequence. Via immobilizing a complementary sequence of cDNA on the test line, the existence of the cDNA (positively correlated to the amount of target) can be detected.

In a more recent study, a TID based Apt-LFA was developed by Ou and colleagues for kanamycin detection, by hybridizing a kanamycin aptamer with a ssDNA sequence partially complementary to the aptamer (this ssDNA is referred to as cDNA herein) 66. According to the design (Figure 12), in the presence of kanamycin, the binding of kanamycin to the aptamer resulted in the desorption of the cDNA. As the amount of the dissociated cDNA is positively correlated to the amount of kanamycin molecules in the sample, the kanamycin amount could be estimated by developing a nucleic acid-based sandwich LFA to detect the dissociated cDNA. As detailed in Figure 12, firstly, two short oligonucleotides complementary to either the 5'- or 3'-end of the cDNA were carefully designed. Then, these two sequences were conjugated to the AuNP reporter and the test line, respectively, to develop a sandwich assay for cDNA detection. Obviously, in the presence of cDNA (equals the presence of kanamycin), a clear AuNP band could be observed. The control line was conjugated with a sequence complementary to the 3'-end of the AuNP-labelled short sequence. After flowing through the control area, the excessive AuNP/oligonucleotide complexes could be detected for the lateral flow validation. As expected, with the competition occurring in the solution (more sensitive than on strip), accompanied by the complementary nucleic acid-based sandwich LFA, a highly sensitive kanamycin detection was achieved. After a 20 min procedure, the recorded LOD reached 4.96 nM, which was much lower than the LOD (309 nM) proposed by the European Commission for kanamycin detection 66. Recently, the potential of this assay was further confirmed by Shima and colleagues, with a LOD of around 65.2 nM for dopamine detection from urine samples 67.

## 4. The LFA membrane and strategies for aptamer immobilization

### 4.1 Nitrocellulose membrane

Although nitrocellulose membrane has been mostly employed for Apt-LFA development, it is worth noting that owing to its complicated and heterogeneous structure, the application of nitrocellulose membrane is affected by the potential towing effect and diffusion phenomena 68. As shown in a recent study, the type, thickness and pore size of the membrane used in LFA determine not only the immobilization efficiency of affinity agents, but also the overall flow rate, directly affecting the performance of the developed LFAs 69. For example, membranes with a smaller pore size, which generally have a longer migration time for aptamer-target reactions, should be considered for aptamers with a relatively low binding capacity or when a longer competition time is desired (e.g. for competitive LFA development) 69. Furthermore, it was also found that the adsorption capacity, the porosity and humidity condition of the membrane directly affected the sensitivity and specificity of the developed LFAs 68. Unfortunately, due to a lack of systematic investigation, the selection of the membrane generally follows the knowledge derived from antibody-based LFA. Given the different physicochemical properties of aptamers and antibodies, the optimised factors (e.g. thickness, pore size) for antibodies may not be suitable for aptamers. Therefore, for optimum apt-LFA performance, the influence factors of the membrane on LFA need to be comprehensively investigated.

### 4.2 Strategies for aptamer immobilization

#### 4.2.1 Non-covalent strategies

Immobilization of aptamers onto membranes or signal reporters (e.g. AuNPs) is an essential procedure for Apt-LFA development. Although the detailed mechanism behind the immobilization of nucleic acids (or proteins) to nitrocellulose membrane (the most exploited membrane) is still unclear, this non-covalent, hydrophobic interaction-mediated binding has been practised since 1960, and constructs the basis of several popular molecular biology techniques such as Southern blotting (nucleic acid immobilization) and Western blotting (protein immobilization). Theoretically, aptamers can be directly immobilized onto nitrocellulose membranes by simply adding aptamer solution to the membrane. However, direct physical absorption is not practicable because of the relatively loose conjugation, which causes desorption of aptamers from the membrane during liquid flow 70. In addition, the random conjugation of aptamers to membranes may affect the tertiary structure of aptamers in an unpredictable manner, and compromise the binding property of the aptamer. Fortunately, although Apt-LFA is a relatively new concept, the development of antibody-based paper assays over the past decades has provided valuable knowledge in this aspect. To overcome the disadvantages associated with physical adsorption, various strategies previously established for antibody immobilization have been exploited.

The most reported non-covalent method for aptamer immobilization is based on the high binding capacity between biotin and streptavidin. Via conjugating biotin molecules at the end of aptamer sequences and incubating the resulting biotinylated aptamers with nitrocellulose membrane-immobilised streptavidin, a strong aptamer/membrane binding could be obtained. However, utilizing streptavidin protein compromises the merits of using nucleic acid-based aptamers to some extent due to the stability issue. To solve this problem, Su and colleagues introduced a microgel mediated immobilization method through coupling aptamers onto poly(N-isopropyl acrylamide) followed by entrapment of the aptamer/microgel conjugates onto the membrane 71. As demonstrated, the aptamer/microgel complex could be steadily entrapped on the membrane due to its large size, without affecting the recognition ability of the aptamer. Other efforts such as the tandem repeating aptamer mediated immobilization was also practised for enhanced aptamer density and improved resistance to nuclease degradation 72.

#### 4.2.2 Covalent bond-based aptamer immobilization

Both physical adsorption and biotin-streptavidin based aptamer immobilization cause uneven immobilization of aptamers onto the membrane surface, and potentially affect the consistency of LFA. In this aspect, the covalent immobilization strategy represents a potential solution. As observed, nucleic acids could covalently bind to the nitrocellulose membrane through ultraviolet (UV) light exposure 73. Indeed, via a 15 min 254 nm UV light exposure, covalent immobilization of aptamers to nitrocellulose membrane could be achieved 69. However, the naked aptamer-mediated immobilization could cause a flat immobilization pattern and potentially affect the binding affinity of the aptamer. To solve this problem, the authors further labelled an amine-C6 linker to the 5'-end of the aptamer to serve as a tethering point. After UV exposure, the aptamer could stand vertically away from the membrane surface and maintain its original structure. Although representing a simplified method, immobilization of aptamers via UV light may induce intramolecular structural changes by thymine dimerization, thereby affecting the reliability of the developed Apt-LFA. As a result, this strategy has rarely been practised in recent years. The aldehyde-amine-based covalent immobilization introduced by Pelton's group is a more practical strategy 74. Firstly, the authors pre-modified the membrane with aldehyde groups. Then, via incubating amine-conjugated aptamers, a covalent immobilization was achieved. Very similar to the streptavidin-biotin based immobilization, this method is featured by both simplicity and reliability. Importantly, such aldehyde-amine based aptamer immobilization offers sufficient structural flexibility and ensures the formation of the intact tertiary structure of the aptamer for reproducible LFA detection. However, it is worth noting that apart from requiring pre-modification of aptamers via extra chemical modifications, systematic investigation of the surface chemistries of the membrane is often required for optimized immobilization results 74.

## 5. Signalling strategies of Apt-LFA

The signalling element is of critical importance for high-quality LFA development. Although AuNP has been widely used in LFA as a signal reporter, the recent progress in material science has provided many new candidate reporters for LFA design, via either visible readout (e.g. liposome-encapsulated dyes, silver nanoparticles, coloured latex beads, carbon) or indirect signal detection (e.g. magnetic particles, Qdots, organic fluorophores, upconverting phosphors). Generally, to be eligible for LFA development, a reporter molecule needs to show features including high stability, ease of modification for recognition probe conjugation, and importantly, without affecting the affinity properties of the immobilized recognition probes 75.

### 5.1 Apt-LFA with colourimetric readouts

The colourimetric change can be easily observed without using any additional instruments, and is therefore preferred for point-of-care LFA development. AuNPs with a diameter of 10-100 nm are the most employed optical indicators for Apt-LFA development and construct the basis of many well-cited Apt-LFAs, due to their high stability, intense visible colour, easy labelling and large-scale production 76. As displayed by the first reported Apt-LFA for thrombin recognition, via incubating thiolated aptamers with AuNPs (Au-S bond), an aptamer-AuNP complex could be produced for signalling purpose 30. However, the application of AuNP is affected by several factors. Firstly, the synthesis of AuNPs associated with expensive procedures. Secondly, the stability of the AuNP is dependent on the electrostatic repulsion between individual particles. Without careful optimization of the buffer system (in terms of ion type, ion concentration and pH) and aptamer concentration, irreversible AuNP coagulation is easy to occur 61. Thirdly, the detection limit of AuNP-mediated visual detection is not satisfactory in many cases, especially for the detection of large particles (e.g. extracellular vesicles or viruses), as the signal intensity of AuNP is negatively affected by the volume ratio of AuNPs to target molecules 77. Consequently, modifications have been made to further improve the sensitivity of AuNP-based LFA. One of these modifications involves the use of enzyme-mediated signal amplification. In a recent study, by immobilizing peroxidase (HRP) onto the surface of AuNPs, Parolo and colleagues designed a high-sensitive LFA facilitated by the commonly used 3,3',5,5'-tetramethylbenzidine (TMB) mediated catalytic signal amplification 78. As reported, up to one order of magnitude of sensitivity could be achieved without losing the simplicity of the LFA. However, as mentioned previously, the employment of enzymes (instability and high cost) could compromise the advantages of employing nucleic acid-based aptamer techniques.

**Figure 13:**
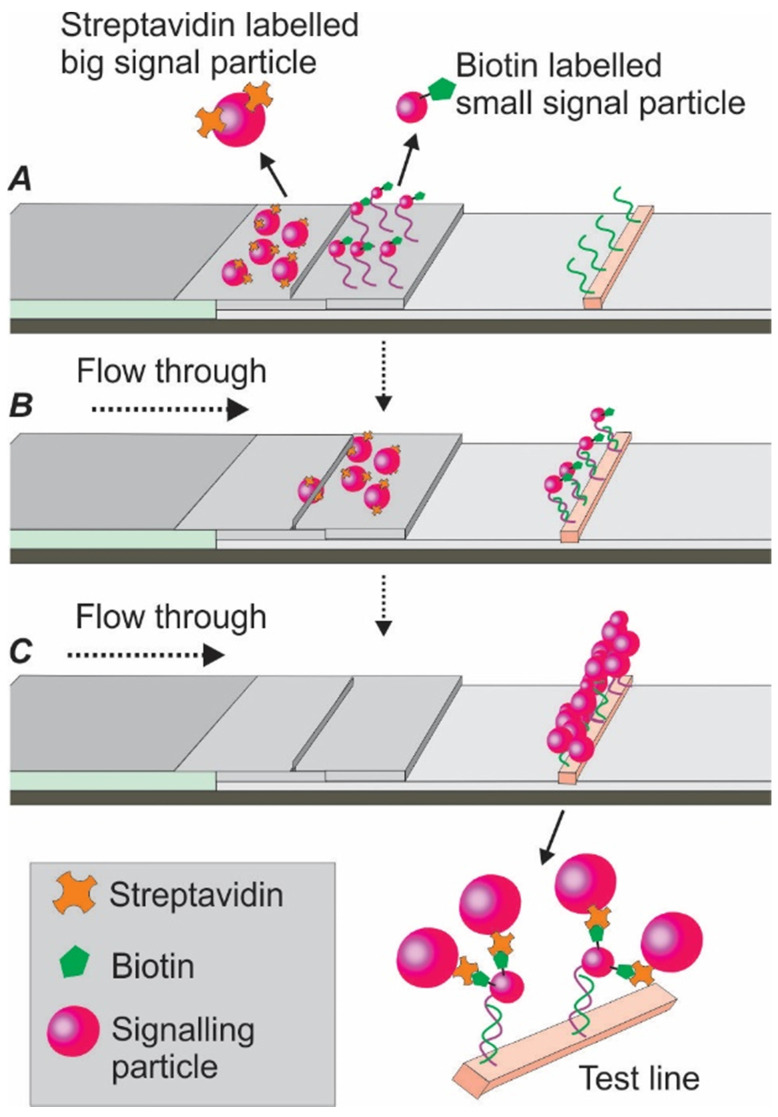
** Schematic illustration of the dual-reporter strategies for enhanced signalling.** (A) the conjugate area consists of two conjugate pads. The first pad is immobilized with streptavidin-labelled bigger reporters while the second pad with biotin-labelled affinity agent /smaller reporter complex. (B) the smaller AuNPs flow faster and bind to the capturing agent on the test line; (C) the bigger reporters flow slower and bind to the smaller reporters (via streptavidin/biotin binding) for enhanced signalling.

The high-sensitive silver staining technique, involving the deposition of metallic silver onto the protein surface, is a simplified and potent signalling strategy. In one study, via using a mixture of silver lactate and hydroquinone, Vasily and colleagues significantly improved the LOD of an AuNP-based LFA by 15 times for potato leafroll virus detection 79. Another way to enhance the optical sensitivity is through the addition of palladium (Pd). Recently, by combined application of AuNPs and Pd, Cheng et al. developed an aptamer nanoflower-based LFA. Via modifying the optical properties of the AuNPs, a dramatic shift of red colour into the near-infrared region was observed. Through a smartphone-based thermal reader application, the authors achieved a 71-fold higher sensitivity compared to the AuNP alone control 14. In another study, to improve the sensitivity of the current AuNP-based LFA, Zhu and colleagues developed a dual-reporter strategy 80. As shown in Figure 13, this assay was characterized by two separate conjugate pads. The first pad was immobilized with streptavidin-labelled bigger AuNPs (41 nm), while the second pad was immobilized with biotin-labelled affinity agent/smaller AuNPs (13 nm) complexes. After adding target-containing samples, the target/affinity ligand/smaller AuNP complexes reached the test line first (due to their smaller size) and were captured by a second capture agent. When the slow running bigger streptavidin-labelled AuNPs later reached the test line and conjugated with the smaller AuNP aggregates via biotin-streptavidin linkage, an improved signal could be detected. As reported, this dual-reporter strategy achieved a LOD of 1 pg/mL for the high-sensitivity cardiac troponin I (hs-cTnl) protein, which was 1000-fold lower than an AuNP-based LFA using antibody 80. Following a similar strategy, in 2016, the same group extended the application of this method to the magnetic nanoparticle-based LFA for highly sensitive and selective carcinoembryonic antigen detection 81.

The magnetic nanoparticle (MNP) represents a promising substitute for AuNP-based visual detection. Similar to AuNPs, MNPs of different size (and magnetite content) show different colour (due to different reflection spectra) 82. However, unlike AuNPs, for which the dispersion can easily be affected by various physicochemical properties such as pH value and salt ions, the highly stable MNP makes the labelling process independent of experimental factors and suitable for various assay matrices. As demonstrated by the dual-reporter strategy-based LFA developed in Zhu's group 81, the utilization of MNPs enabled a LOD of 0.27 ng/ml, comparable to the much more complicated electrochemiluminescence immunoassay (ECLIA) 81. Importantly, the magnetic particle signals could be measured via the magnetic reader (measuring the change of magnetic field) for sensitive and low-background signal detection 83. For example, to improve the currently approved AuNP-based LFA for Ebola virus screening (15% error margin), Yan and colleagues developed a Fe_3_O_4_ magnetic particle-based LFA 84. As demonstrated, with a 30 min operation time, the author achieved a LOD of 1 ng/mL (calculated according to glycoprotein amount), which was around 100-fold more sensitive than the traditional AuNP-based LFA.

In addition to MNP, other materials such as latex beads have also been exploited. In 2013, to detect DNA molecules from plasma, Mao and colleagues developed a nucleic acid hybridization-based sandwich LFA 85. As demonstrated, via using two separate nucleic acid probes and blue dye-doped latex beads, the developed assay system achieved a LOD of 3.75 fmol, similar to that of the AuNP system but with a much lower cost. Indeed, featured by lower cost, high uniformity, reproducibility and compatibility to various chemical modifications, latex beads represent a promising substitute of AuNPs. Notably, latex beads are very suitable for developing multiplexing LFAs, as latex beads with multiple colours are readily available. In a recent study 86, via employing latex beads labelled with two different colours, the authors developed a sandwich LFA for simultaneously detecting IgGs and IgMs of two different viruses in a single assay, opened a new avenue for the development of simplified devices for multiple viruses and/or bacteria detection. The main limitation of latex beads lies in their instability (reduced colour intensity due to leaking) in buffer solutions or when long-term storage is required. In recent years, although the progress in material science has enabled the development of latex beads with enhanced stability, as demonstrated by a recent brucellosis detection study led by Zhu 87, further improvement in terms of signal intensity and stability is still required.

In conclusion, although featured by great simplicity and widely used for POC device development, the nature of visual detection inevitably compromises its sensitivity. This is especially true when high sensitivity is required, such as early-stage virus infection and foodborne pathogens detection (a zero-tolerance of foodborne pathogens is demanded by the food safety testing industry). Over the past decades, various novel non-visual detection approaches have been exploited, including fluorescent imaging and nucleic acid amplification-based Apt-LFA detection.

### 5.2 Fluorescence-based Apt-LFA signalling

Directly conjugating fluorophores onto aptamers is a straightforward method for fluorescence mediated signalling. To detect the faint amount of aflatoxin B1 (AFB1) from food, Chao *et al.* synthesized a cyanidin 5 (Cy5, a fluorophore) conjugated DNA aptamer for competitive Apt-LFA design, with standard AFB1 molecules immobilized on the test line for aptamer competition 88. A semi-quantitative detection was achieved by comparing the fluorescent intensities of the test line and control line. However, although directly conjugating the fluorophore to aptamer obviates the necessity of utilizing extra reporters such as AuNP and latex beads, the direct application of fluorescent dyes is affected by low fluorescent intensities caused by photobleaching and metabolic/chemical degradation.

Research on high-performance fluorescence labelling has led to the development of photo-stable quantum dot (Qdot) based Apt-LFA. First introduced in 1980, Qdots are nano-sized particles (2-10 nm) of a semiconducting material 89. Characterized by unique electronic and optical properties, Qdots display excellent auto-fluorescence, water solubility, and accessibility to various chemical modifications for enhanced signalling 89. Similar to AuNPs, the optical property of Qdot is strictly determined by its size (and shape). It is possible to produce Qdots of any fluorescence colour from the same material by adjusting the dot size, which is useful for multiple targets detection. Over the past decades, Qdots have found their way in broad biomedical applications such as medical imaging and biosensor development 89.

Recently, to improve the sensitivity of foodborne pathogen detection, a sandwich Apt-LFA using red fluorescence-emitting Qdots was developed 69. Briefly, biotin was firstly labelled to the 5'-end of the detection aptamer, followed by conjugating the aptamer to Qdots via streptavidin-biotin binding. To confirm the enhanced detection efficiency of Qdot-based Apt-LFA, a second LFA was prepared by replacing Qdots to AuNPs. Notably, facilitated by a portable 365nm UV lamp, the signal could be visualized by naked eyes. The subsequent comparison revealed that the Qdot-based LFA obtained a 10-fold higher sensitivity than that of the commonly used AuNP-based LFA (6000 *E.coli*/mL vs 600 *E.coli*/mL). Importantly, this high-sensitive detection could be further improved by employing an orange glass filter. Later, following a similar procedure, another Qdot-based LFA was developed by Wilkins and colleagues for N-terminal pro B type natriuretic peptide (NT-proBNP, cardiac biomarker) detection 90. As demonstrated, via using blue coloured Qdots as reporters, the developed LFA was able to detect NT-proBNP with high specificity and sensitivity, ready for clinical translation. In another study, by conjugating an ochratoxin A (OTA) specific aptamer to Qdots, Xu and colleagues reported a Qdot-based semi-quantitative Apt-LFA 50. As shown, through comparing the fluorescent intensity of the Qdots in the test zone and control zone, the authors achieved a LOD of 1.9 ng/mL, meeting the minimal limitation requirement of OTA detection in wines and juices as issued by the European Commission Regulation 50.

However, despite their small size, Qdots show poor migration (due to clumping) in nitrocellulose membrane 91, which represents a potential obstacle for Qdot-based LFA development. To solve this problem, a comprehensive comparison of the commonly used reagents (e.g. surfactant) for particle suspension was performed by Berlina and colleagues; it was found that adding high concentration (i.e. 5%) of Tween 20 could dramatically improve the mobility of Qdots on nitrocellulose membrane without compromising the detection efficacy, similar to earlier observations 92.

In addition to replacing AuNPs, a combinatorial application of AuNPs and label-free Qdots has been proven to be a plausible method for enhanced LFA design, as displayed by an avian influenza virus detection work led by Li and colleagues 93. Differing from the commonly used virus detection strategy (detecting virus-initiated IgG and/or IgM) which saw a high probability of false-negative results (virus-initiated IgG or IgM takes up to 2 weeks to develop), the authors developed an AuNP-based LFA for more reliable virus detection by directly analysing the existence of protein markers of viruses. However, although this method enables direct virus detection, the relatively low amount of virus particles in body fluid, especially at the early stage of infection, puts forward to the requirement for ultrasensitive signal readout. To address this issue, the authors firstly developed an AuNP-based sandwich LFA. Once the AuNPs aggregated at the test zone (in the presence of the virus), the AuNPs were dissolved in 0.15 M hydrogen chloride (HCl) and 0.8 M bromine (Br_2_) to release gold ions, which were then transferred to a Qdot-containing microplate. As the gold ions have been confirmed to be able to quench the fluorescence of Qdots in a concentration-dependent manner 94, the concentration of the gold ions, and therefore the intensity of the AuNP band on the test zone, could be calculated. Surprisingly, by transferring the visual signal of AuNP to Qdot-based fluorescence analysis, the authors achieved a LOD of 0.09 ng/mL, up to 100-fold more sensitive than the original AuNP-based visual detection.

Apart from Qdots, the lanthanide-doped upconverting nanoparticle (UCNP), capable of converting near-infra-red excitation into the high energy visible region (hence its name: upconverting), represents another promising material for fluorescence-mediated LFA design. Differing from other fluorescent molecules, the UCNPs do not display autofluorescence and photodegradation because of the infra-red region excitation, which constructs the basis for its wide applications ranging from deep-tissue bioimaging, nanomedicine, fluorescent microscopy, volumetric display to security labelling 95. Recently, for high-sensitive OTA detection, Wang and colleagues introduced a UCNP-based competitive Apt-LFA 96. As shown in this study, an OTA specific aptamer was conjugated to UCNP via a carboxyl linker. As the binding of OTA to the aptamer/UCNP complex competed for the binding between the aptamer and its partially complementary ssDNA sequence on the test zone, a semi-quantitative OTA detection could be obtained by calculating the relative fluorescence intensity (test line value/control line value, excited by a 980 nm laser). With a LOD of 1.86 ng/mL in wheat and beer samples, this device displayed a similar sensitivity with a previously reported Qdot-based LFA 97. In addition to small-molecule detection, the UCNP-based Apt-LFA was also useful for the detection of protein markers. Recently, Ali M *et al.* developed a sandwich Apt-LFA using UCNP mediated signalling for visceral adipose tissue-derived serine protease inhibitor (vaspin, an obesity and type 2 diabetes marker) detection 98. With only 3 minutes processing time, this assay achieved a LOD as low as 39 pg/mL, more than 15 times higher than that of the previously reported AuNP-based Vaspin LFA 31. However, as reported, the product and labelling procedures of UCNP could be laborious, and the reproducibility needs to be further improved to reduce the high batch-to-batch variation associated with UCNP-based LFA 31.

### 5.3 Aptamer-gated fluorophore nanoprobes

The recently developed aptamer-gated fluorophore detection represents another promising fluorescence signalling strategy. Theoretically, any aptamer can be modified with a hairpin structure to serve as a gate to trap fluorescent molecules into a porous particle 99, 100. Upon target recognition, the gate can be opened as a result of the conformational change of aptamers, which leads to the release of the blocked fluorophores for signalling purposes. In a recent work, Ozalp and colleagues reported an aptamer-gated silica nanoprobe-based LFA for ATP detection 99. Firstly, according to a universal approach for gating aptamer design 100, the authors designed an ATP aptamer ended with a hairpin structure by adding a short sequence that was partially complementary to the 3'-end of the original aptamer. Then, the mesoporous silica particles, a widely used drug delivery material featured by large pore volume, were fabricated with rhodamine B (a fluorophore molecule) blocked inside the pores (via immobilizing the gating aptamer near the mouth of the pore). After that, the aptamer-gated fluorophore-loaded silica nanoparticles were immobilized onto the test line of the LFA. After ATP containing samples migrated from the sample pad to the test line, the binding between ATP and the aptamer could change the intramolecular conformation of the gating aptamers and disrupt their hairpin structure 101, which opened the silica nanopores (allowed the release of the captured fluorescent molecules) and resulted in reduced fluorescence signal in the test line. Because the release rate of the fluorophores was proportional to the concentration of target molecules in the solution 102, by quantifying the fluorescent signal on the test line before and after the LFA reaction, the concentration of the ATP molecules in the tested samples could be estimated.

Different from the commonly used LFA, the signalling mechanism of the aptamer-gated nanoprobe LFA relies entirely on the structural change of the aptamer upon target binding, without the requirement of a second aptamer sequence (as in sandwich assay) or immobilization of target analytes (which involves the difficulty in immobilization of unstable or hard-to-immobilized targets) or partially complementary sequences on the test line (involve the tricky step for complementary sequence design). This makes the aptamer-gated fluorescence detection a promising approach for simplified LFA design. And importantly, although previous efforts focused mainly on small molecule detection, as gating aptamers could be designed from aptamer sequences of any target, this strategy would be equally useful for detecting large molecules such as proteins, peptides or even whole microorganisms.

### 5.4 Signal amplification via nucleic acid amplification

Apart from increasing the signal intensity via fluorophores, the nucleic acid nature of aptamer enables developing high-sensitive LFA via exploiting many of the established nucleic acid amplification techniques 22, 103. Although PCR represents a standard method for nucleic acid amplification, the requirement of multiple procedures, reagents, and additional apparatus makes it impractical for LFA development. For practical LFA design, nucleic acid amplification strategies with more simplified procedures have been explored. The recombinase polymerase amplification (RPA) is such an example. As a type of isothermal reaction (reacting under constant temperature), RPA could be performed even in a water bath, making it an excellent candidate for developing low-cost and rapid POC tests 104. In a recent study, an RPA facilitated competitive Apt-LFA was reported by Jauset-Rubio and colleagues for β- conglutin detection 56. Differing from the common LFAs where the competition takes place on the membrane, to facilitate RPA-based signal amplification, the key competition step was designed to take place in a tube, where magnetic-bead conjugated β-conglutin molecules compete with β-conglutin protein in the test samples for aptamer binding. After incubation, aptamers bound to the bead-immobilized β-conglutin were amplified via RPA reaction for 15min. As the primers used in RPA were featured by a tail end, all amplicons were flanked by ssDNA tails on 5' and 3' ends, which enabled the design of a sandwich LFA, via labelling a detection oligonucleotide (complementary to the 5' tail of the amplicon) onto AuNPs, and immobilizing a capture oligonucleotide (complementary to the 3' tail of the amplicon) onto the test line. When compared with the ordinary competitive Apt-LFA using identical sets of aptamer and target (LOD=55 pM), this RPA-based LFA (9 fM LOD) showed a more than 6,000-fold sensitivity. Although this RPA-based Apt-LFA facilitated high-sensitive target detection, there are concerns associated with its application. Firstly, as a type of competitive assay (the signal strength is negatively associated with the amount of target molecules in the samples), the presence of the target has to be assessed by comparing the band density of the test line with a target-free control. Though RPA is acceptable for normal competitive LFA, the systematic deviation of this multi-step assay could be dramatically magnified by various factors including the amplification bias, the error during aptamer elution, and the non-specific binding between the magnetic beads and aptamers, resulting in unwanted false negative/positive results. Secondly, the application of protein enzyme inevitably compromises the merits of using nucleic acid aptamers, as previously discussed. Alternatively, enzyme-free nucleic acid amplification techniques such as hybridization chain reaction (HCR) 105 and catalysed hairpin assembly 106 have also been exploited. Indeed, featured by a one-step procedure, ease to scale up, and no requirement for perishable protein materials, these enzyme-free strategies hold great potential for novel functional nucleic acid-based LFA design. For instance, via using HCR technique, a high-sensitive DNAzyme-based LFA for Pb^2+^ detection was developed by Chen and colleagues in 2013 105. In this work, a carefully designed substrate sequence was firstly hybridized with a DNAzyme sequence (the recognition agent for Pb^2+^ targets). Upon target recognition, the cleavage of the substrate sequence (by the DNAzyme) served as an input to trigger the HCR to produce biotin-tagged sequences. As these biotinylated sequences contain cohesive ends that could hybridize with a ssDNA probe, via conjugating the ssDNA probe on AuNPs, a sandwich LFA with LOD as low as 10 pM was developed. Although DNAzyme was used in this assay, as previously commented by a *Nature* publication, “If we succeed in developing a general aptamer triggering mechanism, then HCR amplification could be incorporated in sensors for a wide range of small molecules” 107. Indeed, developing HCR triggers by modification of existing aptamers would dramatically improve the sensitivity of existing Apt-LFA.

## 6. Conclusion and perspectives

Since the development of the first LFA by Unipath in 1985, different types of LFAs have been reported for the detection of a wide range of target analytes including pathogens of infectious diseases (e.g. virus infection, malaria), toxins (e.g. endotoxins, snake venom), environmental pollutants (e.g. antibiotics, pesticides, heavy metals), small molecule drugs (e.g. cocaine) and metabolites (e.g. ATP) 133. However, despite huge market potential and popularity, the conventional LFA faces challenges such as batch-to-batch variation and instability 133. This is mainly due to the fact that conventional LFAs rely heavily on animal-derived antibodies, which display inter-batch variation due to the physiological variation among animals.

The rapid progress in aptamer (chemical antibodies) technologies over the past three decades provide a valuable opportunity to solve many of the obstacles faced by the conventional antibody-based LFAs 53, 134. Aptamers are identified via *in vitro* procedures which do not require the use of animals, and compatible with chemical modifications 11. This not only allows developing aptamers against non-immunogenic molecules (i.e. metal ions) or toxic molecules (e.g. toxins) that are not feasible for animal-based antibody development, but also, allows exploiting various nucleic acid-based analytical techniques (e.g. displacement assays, non-enzyme amplification techniques). Importantly, while the application of antibodies is limited to close to physiological conditions, aptamers could be selected and applied in non-physiological conditions akin to the real-world application (e.g. detecting reducing agents). These features, together with high affinity, small size, superior stability, ease of synthesis and freedom to incorporate chemical modifications, collectively make aptamers excellent recognition agents for biosensor development 135. Furthermore, the aptamer technique allows identification of targets even when detailed knowledge about the target is not available. The recently developed Cell-SELEX is an example 136. By employing intact cells as targets, coupling with optimized selection controls, Cell-SELEX allows researchers to identify aptamers to target cells in the absence of information about their surface structure which is very useful for the detection of new infectious diseases. Accordingly, aptamer industry reaches 245 million USD by 2020 with a compound annual growth rate of ~17.9% and there is a huge potential for the application of aptamers in LFA for POCT development 137.

However, it should be noticed that changes in assay conditions such as metal ions, buffer system and pH value could dramatically affect the binding property of aptamers and cause reduced detection efficiency 138, 139. As demonstrated by a recent study, when an aptamer-based sensor was tested under different settings, compared with PBS (where the aptamer was identified), the binding affinity of the aptamer could be reduced significantly from 32.49 nM to 1964.4 nM in 50% beer 140. Consequently, incorporating chemical modifications (e.g. 2'-amino pyrimidine, 2'-fluoropyrimidine, 2'-O-methyl and locked nucleic acid) to stabilize the tertiary structure of aptamers, or developing aptamers under the intended assay conditions rather than the commonly used buffer systems (e.g. PBS) are highly recommended 11.

Based on the nucleic acid nature of aptamer, various types of competitive Apt-LFAs have been introduced. Among them, the target molecule (on the test line) mediated aptamer competition is generally preferred. However, it does come with additional costs (for target immobilization) and potential stability issues (depending on the nature of the target). It is worth mentioning that many of the published competitive Apt-LFAs involve the integration of additional bases at the end of the aptamers, with complementary sequences of the additional bases immobilised on the control line for assay validation. However, these tails not only potentially interrupt the binding capacity of the aptamer and resulting in reduced detection efficiency, but also raise concerns for control line design of the competitive Apt-LFA, especially when a low amount of aptamers were immobilized on the conjugate pad. In such a case, when the amount of targets was low in the sample, all the aptamers may bind to the immobilised targets or oligonucleotides, with no aptamer available for control line binding (results in invalid assay control). In contrast, a high target concentration may cause all the aptamers being occupied by target molecules in the sample and again, no aptamer would be available for control line binding. Therefore, for effective competitive Apt-LFA development, the amount of aptamers, competitive targets/competitive oligonucleotides, as well as the complementary sequences in the control line have to be experimentally tested 10. In fact, as previously mentioned, for more effective experimental control, full complementary sequences of the aptamers could be immobilized at the control line for aptamer recognition.

Although aptamers are suitable for detecting small molecules which are not accessible to antibody development, to our knowledge, only limited efforts have been made in this area, with Apt-LFAs reported only for the detection of AFB1, E2, bisphenol A (BPA), ATP, p-amino hippuric acid, Ochratoxin A, dopamine, ampicillin, cocaine and mercury. This is mainly because of the difficulty in developing high-affinity and specific aptamers for small molecule recognition. Despite novel strategies such as the crosslink mediated reporter aggregation (Figure 11) and the cross-recognition aptamer-based detection 58 have shed light on this area, further investigation is still imperative for efficient small molecule detection. This is especially true given the growing interest in the small endogenous metabolites-based disease diagnosis.

As discussed above, the sandwich format is preferred for Apt-LFA development, with one aptamer immobilised at the test line and an additional aptamer linked to a signal reporter for target detection. However, in many cases, developing a pair of aptamers targeting different sites of a target is difficult, especially for small molecules with limited binding motifs. Although the combined application of aptamers and antibodies offers opportunities for sandwich format LFA development, the inclusion of costly and less stable antibodies compromises the inherent advantages of aptamer-based LFAs. Applying split aptamers is an alternative solution for sandwich LFA design when only a single aptamer is available. However, splitting an aptamer into two separate fragments causes reduced binding properties as recorded in our recent LDL-R aptamer development 21. As a result, selecting a pair of aptamers targeting different sites of the target is highly desirable for efficient Apt-LFA development. To this end, strategies such as employing high-efficiency initial libraries or blocking aptatopes (aptamer binding sites) have been suggested 11. The recent integration of next-generation sequencing and computer-based machine learning approaches for aptamer identification also provides a great opportunity for efficient aptamer identification, by in-depth evaluation of the binding motif of aptamers against the target of interest 11.

Whilst various types of Apt-LFA have been developed over the past decades, it should be noticed that a commercial Apt-LFA device is still not available. The relatively low LOD can be the main reason for this shortfall. Although novel strategies such as integrating isothermal amplification techniques and fluorescent readers can dramatically improve the detection sensitivity of LFA, the involvement of additional procedures significantly compromises its simplicity. Therefore, further advances in reducing the complexity of the current signalling readout approaches are required to develop highly sensitive Apt-LFAs. Towards this, a smartphone-based portable quantitative Apt-LFAs powered by the ever-progressing telecommunication techniques and integrated image processing applications hold great potential for the development of novel Apt-LFAs with improved efficiency.

## Figures and Tables

**Figure 4:**
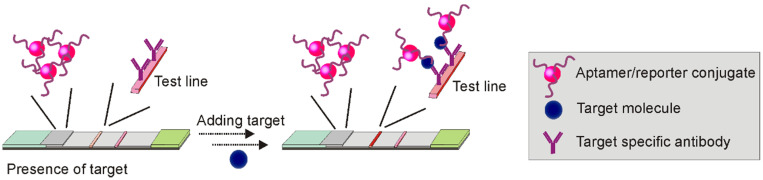
Schematic illustration of Apt-LFA using a combination of aptamer and antibody.

**Table 1:** Representatives of aptamer-based lateral flow assays

Format	Target	Limit of detection	Sample matrix	Reporter	Ref.
**Sandwich assay**					
**Aptamer pair-based sandwich assay**					
	β-conglutin	8 fM	PBS	Fe_3_O_4_@AuNPs	17
	Creatine kinase MB	0.63 ng/mL (7.5 pM)	Artificial serum	Fluorescent sphere	15
	Exosomes	1.4 x 10^4^ exosomes/μL	PBS	Au@Pd Nanopopcorn	14
	Salmonella Typhimurium	10^3^ CFU/mL	PBS	AuNPs	108
	Escherichia coli O157:H7	10^4^ CFU/mL	PBS	AuNPs	108
	Chikungunya virus, Tick-borne encephalitis virus	-	PBS	Magnetic beads	33
	Staphylococcus aureus	10^4^ CFU/mL	PBS	AuNPs	108
	*E.coli*	300 cells	PBS	Qdots	69
	Ramos cells	800 cells (strip reader)	PBS	AuNPs	34
	Thrombin	2.5 nM	NaCl-Na_3_C_6_H_5_O_7_ buffer	AuNPs	30
	Vaspin	39 pg/mL (0.86 pM)	PBS	UCNPs	98
	Rongalite	1 μg/mL (8.47 µM)	PBS	AuNPs	109
	Avian influenza H5N2 whole virus particles	1.27 × 10^5^ EID50/mL (PBS) and 2.09 × 10^5^ EID50/mL (duck's feces)	PBS, duck's feces	AuNPs	110
	Platelet-Derived Growth Factor-BB (PDGF-BB)	1.0 nM	PBS	AuNPs	111
	Thrombin	1.5 nM	PBS	AuNPs	111
	Thrombin	0.85 nM	PBS	AuNPs	112
	Human odontogenic ameloblast-associated protein	8.32 nM (PBS), 14.59 nM (saliva)	PBS, Human saliva	AuNPs	113
	Vaspin	0.137 nM	Tris buffer	AuNPs	31
**Aptamer and antibody-based sandwich assay**					
	IgE	0.13 ng/mL (0.65 pM)	PBS	HRP	114
	α-amylase (sAA)	-	Saliva	AuNPs	35
	Thrombin	0.25 nM	NaCl- Na_3_C_6_H_5_O_7_ buffer	Dual AuNP conjugation	115
	Cholera toxin	10 ng/mL (0.387 nM)	PBS	AuNPs	18
	Platelet-derived growth factor BB (with antibody)	1 ng/mL (41.15 pM)	Serum	AuNPs	116
	Human osteopontin	0.1 ng/mL (1.67 pM)	PBS	AuNPs	117
	Type-B aflatoxins	0.16 ng/mL (0.51 nM)	HEPES	fluorescent dye Cy5	118
**Split aptamer-based sandwich assay**					
	ATP	0.5 µM	Urine	AuNPs	42
**Competitive assay**					
**Complementary sequences immobilized on the test line**					
	Progesterone	5 nM	PBS	AuNPs	119
	Aflatoxin B1	0.03 ng/mL (96 pM)	PBS	UCNPs	52
	Zearalenone	10 ng/mL (31.4 nM)	PBS	AuNP & peroxidase	120
	Listeria monocytogenes	53 cells/mL	Minced chicken	aptamer-gated silica nanoparticles	121
	Chloramphenicol	0.5 μg/mL (1.53 µM, visual), 0.0634 μg/mL (196 nM, quantitative)	PBS	AuNPs	122
	Aflatoxin B1	10 ng/mL (32 nM, visual), 1.05 ng/mL (3.4 nM, quantitative)	PBS	AuNPs	122
	OTA, Hg^2+^, Salmonella, HBV,ST-2	-	PBS	UCNPs	123
	OTA	1.86 ng/mL (4.6 nM)	Beer, wheat	UCNPs	96
	Exosome	6.4 × 10^9^ particles/mL	Cell culture medium	AuNPs	124
	Ampicillin	2.71 ng/mL (7.76 nM)	Water	Fluorescent dye HEX	125
	Ochratoxin A (OTA)	4.7 nM	Tris buffer	Qdots	50
	Ochratoxin A (OTA)	0.45 nM (strip reader)	PBS, Wine	AuNPs	126
	Ochratoxin A (OTA)	1 ng/mL (2.48 nM)	PBS	AuNPs	127
	Hg^2+^	0.13 ng/mL (0.65 nM)	Water	Fluorescence	51
**Target molecules immobilized on the test line**					
	Kanamycin	50 nM (visual), 4.96 nM (quantitative)	PBS	Magnetic microspheres	66
	Chlorpyrifos	0.73 ng/mL (2.08 nM)	PBS	Qdots, AuNS	128
	Diazinon	6.7 ng/mL (22 nM)	PBS	Qdots, AuNS	128
	Malathion	0.74 ng/mL (2.24 nM)	PBS	Qdots, AuNS	128
	Mercury ions	5 ppb	PBS	UCNPs	16
	OTA	3 ng/mL (7.4 nM)	PBS	UCNPs	16
	Salmonella	85 CFU/mL	PBS	UCNPs	16
	β‑Conglutin	9 fM (&sequence amplification)	PBS	AuNPs	56
	Cholera toxin	51 ng/mL (1.82 nM)	PBS	AuNPs	18
	Ampicillin	130 pmole	PBS	AuNP	58
	Aflatoxin B1	0.1 ng/mL (0.32 nM)	Food, feedstuff	Cy5	88
**Aptamers immobilized on the test line**					
	CA125	3.71 U/mL	Serum	AuNPs (DAB)	47
**Strand displacement & Target induced dissociation**					
	HER2	20 nM	Human serum	AuNPs	62
	Cortisol	1 ng/mL (~2.7 nM)	Artificial sweat	AuNPs	64
	Ochratoxin A	6.3 nM	PBS	AuNPs, AgNPs	63
	Dopamine	65.2 nM	Urine	AuNPs	67
	Cocaine/Adenosine	20 µM Adenosine; 10 µM cocaine	Tris acetate buffer	AuNPs	65
	Aflatoxin B1	0.32 nM	Corn sample	Cy5 dye	129
	ATP	69 µM	PBS	Rhodamine B dye	99
	*E.coli*	10 CFU/mL	PBS	AuNPs-DNA probe	130
	Salmonella	10 CFU/mL	PBS	AuNPs	131
	Thrombin	6.4 pM	NaCl- Na_3_C_6_H_5_O_7_	AuNP/HRP	132
